# Opioids and Their Endocrine Effects: A Systematic Review and Meta-analysis

**DOI:** 10.1210/clinem/dgz022

**Published:** 2019-09-12

**Authors:** Friso de Vries, Mees Bruin, Daniel J Lobatto, Olaf M Dekkers, Jan W Schoones, Wouter R van Furth, Alberto M Pereira, Niki Karavitaki, Nienke R Biermasz, Amir H Zamanipoor Najafabadi

**Affiliations:** 1 Department of Medicine, Division of Endocrinology, Leiden University Medical Center, Leiden, The Netherlands; 2 Centre for Endocrine Tumors Leiden (CETL), Leiden University Medical Center, Leiden, The Netherlands; 3 Department of Clinical Epidemiology, Leiden University Medical Center, Leiden, The Netherlands; 4 Walaeus Library, Leiden University Medical Center, Leiden, The Netherlands; 5 Department of Neurosurgery, Leiden University Medical Center, Leiden, The Netherlands; 6 Institute of Metabolism and Systems Research, College of Medical and Dental Sciences, University of Birmingham, Birmingham, UK; 7 Centre for Endocrinology, Diabetes and Metabolism, Birmingham Health Partners, Birmingham, UK; 8 Department of Endocrinology, Queen Elizabeth Hospital, University Hospitals Birmingham NHS Foundation Trust, Birmingham, UK

**Keywords:** opioids, analgesics, hypogonadism, hypocortisolism, pituitary

## Abstract

**Context:**

The increased use of opioids has resulted in an unprecedented opioid epidemic. Chronic opioid use causes hypogonadism, but its frequency, as well as the effects of opioids on other hypothalamo–pituitary–end organ hormone axes, remains unclear.

**Objective:**

The aim of this systematic review and meta-analysis was to assess the effects of opioid use on pituitary function.

**Methods:**

Eight electronic databases were searched for articles published up to May 8, 2018. Fixed or random effects meta-analysis was performed to estimate pooled proportions with 95% confidence intervals (CI). This study is reported following the PRISMA and MOOSE guidelines.

**Data synthesis:**

52 studies (22 low risk of bias) were included describing 18 428 subjects, consisting of patients with chronic pain (*n* = 21 studies) or on maintenance treatment for opioid addiction (*n* = 9) and healthy volunteers (*n* = 4). The most frequently used opioid was methadone (*n* = 13 studies), followed by morphine (*n* = 12). Prevalence of hypogonadism was 63% (95% CI: 55%–70%, 15 studies, 3250 patients, 99.5% males). Prevalence of hypocortisolism relying on dynamic and nondynamic testing was 15% (95% CI: 6%–28%, 5 studies, 205 patients, 57.5% males) and including only studies using the insulin tolerance tests 24% (95% CI 16%–33%, 2 studies, *n* = 97 patients). In 5 out of 7 studies, hyperprolactinemia was present. No clear effects on the somatotropic and hypothalamo–pituitary–thyroid axes were described.

**Conclusions:**

Hypogonadism occurs in more than half of male opioid users, and hypocortisolism in approximately one-fifth of all patients. Periodical evaluation of at least the gonadal and adrenal axes is therefore advisable.

Over the past two decades, the use of opioids and the number of opioid overdose-related deaths has steadily increased (1). In the United States alone, there were more than 11 million people with misused prescription opioids and 42 000 opioid-related deaths were reported in 2016 (2–4). Long-term opioid use is associated with adverse effects, the most common being constipation, nausea, and dyspepsia (5, 6). In addition, several studies suggest that the endocrine system is affected in opioid users (7, 8).

In addition, many animal studies have been performed on the mechanisms of opioid-induced endocrine effects. It has been shown that opioids inhibit the gonadal axis in the hypothalamus via ε-receptors and stimulate prolactin secretion via μ-, κ-, and δ-receptors. Thus, gonadotropin-releasing hormone (GnRH) release and the gonadal axis may be additionally suppressed by opioid-induced hyperprolactinemia (2). Lastly, opioids induce the conversion of testosterone to dihydrotestosterone (9). The corticotropic axis may be modulated via effects on the κ- and δ-receptors in the hypothalamus and pituitary gland and the somatotroph axis via μ-, κ-, and δ-receptors in the hypothalamus (2).

Although hypogonadism and, to a lesser extent, hypocortisolism are recognized endocrine side effects, their prevalence remains unclear (10, 11). Dysfunction of both axes may result in significant, often incapacitating symptoms (2). Both male and female patients with hypogonadism may suffer from sexual dysfunction and decreased libido. Male patients can present with erectile dysfunction, impotence, and gynecomastia, while female patients can have menstrual irregularities. In addition, hypocortisolism can manifest a wide variety of symptoms, such as fatigue, malaise, abdominal discomfort, anorexia, and orthostatic hypotension. Possible effects of opioids on the secretion of growth hormone (GH), thyroid-stimulating hormone (TSH), and prolactin remain unelucidated and have not been systematically reviewed (2, 12–17).

Due to the increased use of opioids, it has become increasingly important to identify the prevalence and impact of opioid exposure-related endocrine deficits. Our goal was, therefore, to assess the reported effects of opioids on the endocrine system through a systematic review and meta-analysis.

## Materials and Methods

This systematic review and meta-analysis was reported according to the Preferred Reporting Items for Systematic Reviews and Meta-Analysis (PRISMA) statement (18) and the Meta-analyses of Observational Studies in Epidemiology (MOOSE) guideline (19) for randomized and observational studies, respectively. Screening of studies, data extraction, and risk of bias assessment were performed by 2 independent reviewers (FdV, MB). Disagreement was solved through discussion. If discussion failed to lead to a consensus, a third reviewer was consulted (AHZN) to reach consensus.

### Search strategy

A literature search was conducted to identify studies describing endocrine effects of opioid use. The following databases were systematically searched for relevant studies with help of an experienced librarian (JWS): PubMed, Embase, Web of Science, COCHRANE Library, Emcare, Academic Search Premier and ScienceDirect. Data were colleted up to May 8, 2018 and processed to an EndNote X9 database (Clarivate Analytics, Philadelphia, PA, US). Terms or derivatives of these terms included in the search strategy were “opioids,” “hypogonadism,” “adrenal insufficiency,” “growth hormone deficiency,” “hypothyroidism,” and “prolactin.” Furthermore, terms formulated to exclude animal studies were used. The reference lists of included studies were reviewed to identify additional relevant studies. The complete search strategy is presented in a supplemental document 1 (20).

### Eligibility criteria and article selection

Randomized controlled trials, longitudinal, and cross-sectional cohort studies assessing the endocrine status in patients using opioids were eligible for inclusion. Studies were excluded if the study population (partially) consisted of children (age < 18 years) or if studies were not in English. Additionally, studies not reporting original data (eg, reviews), case reports, and unpublished studies (eg, congress abstracts) were excluded from analysis. Studies were screened by title and abstract and potentially relevant studies were reviewed by full-text analysis.

### Data extraction

The following data was extracted, if available: main inclusion and exclusion criteria, number of included subjects, age, percentage of male subjects, opioid type, duration of opioid exposure, duration of follow-up, endocrine axis (including evaluation method), and the effects of opioid exposure on the described axes. Where possible, the number of patients with endocrine dysfunction(s) were extracted. Finally, results of hormone replacement therapies in case of presumed endocrine deficiencies were extracted when available. Twenty authors of studies reporting on the function of the hypothalamo– pituitary–gonadal and hypothalamus–pituitary–adrenal (HPA) axis were contacted and asked to provide additional data on the prevalence of these respective deficiencies. In case of no response, authors were approached a second time; however, ultimately no additional (subject level) data could be obtained.

### Risk of bias assessment

The following risk of bias components were assessed among all studies:

1. Consecutive inclusion of patients
2. Adequate endocrine testing
3. Risk of confounding of effects of opioids on the endocrine system among comparative studies (eg, comparison of opioid addicts and healthy controls, correction with multivariate analyses)

Studies were assigned to qualitative categories for each element of the risk of bias analysis and were defined as low, moderate, or high risk for each element separately (the scoring system is presented in the supplemental document 2 (20). Adequate endocrine testing was considered the most important element of the final risk of bias classification. Studies with a low risk of bias on the endocrine assessment and at least 1 of the other 2 criteria were assigned a low risk of bias. Potential differences between studies were used to assess the between-study heterogeneity. This was largest in endocrine assessment of the HPA axis, and, therefore, we performed a separate assessment based on studies using the insulin tolerance test (ITT).

### Study endpoints

The primary outcome measure was the percentage of patients with dysfunction of 1 or more pituitary axes (see supplemental tables S1–S6 for the reported definitions of endocrine dysfunctions) (20). Furthermore, we systematically reviewed the effects of opioids on the various hypothalamic–pituitary–end organ axes (gonadal, adrenal, thyroid, somatotroph, and prolactin secretion), as well as the effects of hormone replacement on opioid-related endocrine deficiencies.

### Statistical analysis

A random effects logistic regression model was performed when there were 5 or more studies per analysis to estimate pooled percentages. A fixed-effects model was used for analyses with fewer than 5 studies. To prevent exclusion of studies with a 0% or 100% outcome, the Freeman–Tukey arcsine transformation was used to stabilize variances. For outcomes that included 5 or more studies per analysis, I^2^ statistics were used for the quantification of between-study heterogeneity. For analysis with less than 5 studies, no quantification of between-study heterogeneity was estimated due to reliability issues (21). All analyses were performed using Stata 14 (Stata Corp., College Station, TX, US). Sensitivity analysis for the gonadal axes was performed by analyses of studies with a low risk of bias. For the meta-analysis of studies that analyzed the HPA axis, sensitivity analysis was performed including only studies that used the ITT as assessment method.

## Results

### Study selection

**Figure 1.**
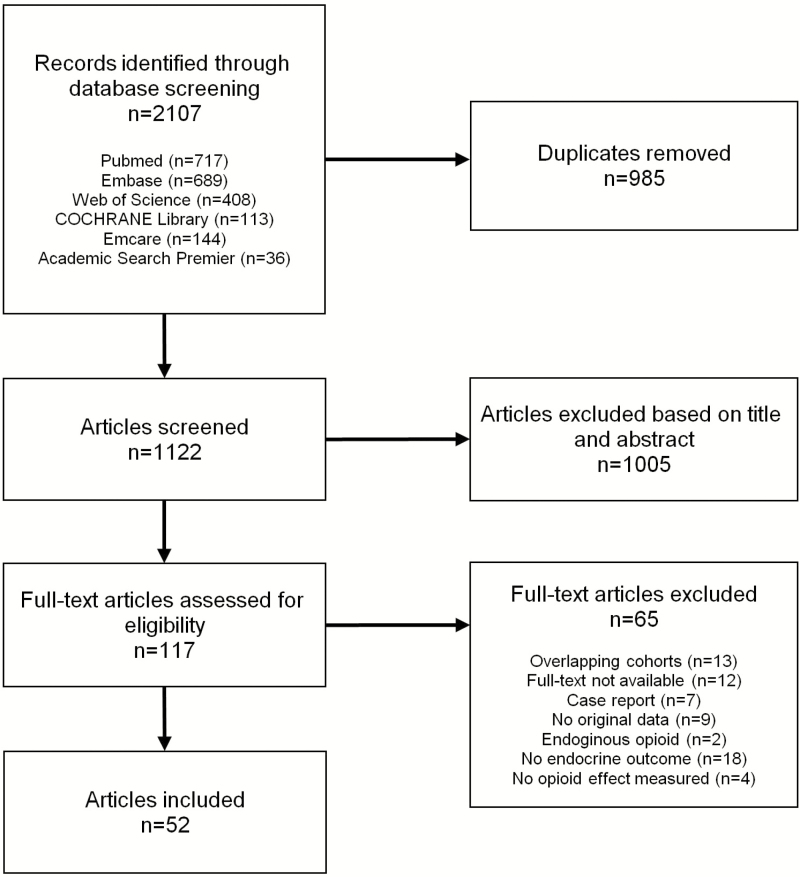
Selection and screening of studies.

The literature search yielded 1123 unique articles. After the exclusion of studies based on title and abstract, we screened 118 full-text articles. Ultimately, 52 studies were included (Fig. 1) (20). Of all studies, 22 were classified with a low risk of bias, 10 with a moderate, and 20 with a high risk of bias. The full risk of bias assessment is presented in supplemental table S7 (20).

### Study characteristics

Of the 52 included studies, 34 analyzed the effect of opioids on the gonadotropic axis, 24 on the HPA axis, 8 on the hypothalamo–pituitary–thyroid (HPT) axis, 9 on prolactin secretion, and 5 on the somatotropic axis. In addition, 6 studies reported on the effect of testosterone replacement in case of associated hypogonadism and 1 on the effect of treatment with hydrocortisone on hypocortisolism, but there were no studies reporting on the effects of hormone replacement on other axes. Studies were published between 1970 and 2018; 32 (62%) were published after 2010. Methadone was the most frequently reported opioid (*n* = 13), followed by morphine (*n* = 12). Eight studies defined the opioid dose as the morphine equivalent daily dose. Most studies were performed in the United States (*n* = 18), Europe (*n* = 18), or Australia (*n* = 6).

### Meta-analysis of HPA axis and gonadal axis deficiency

**Figure 2.**
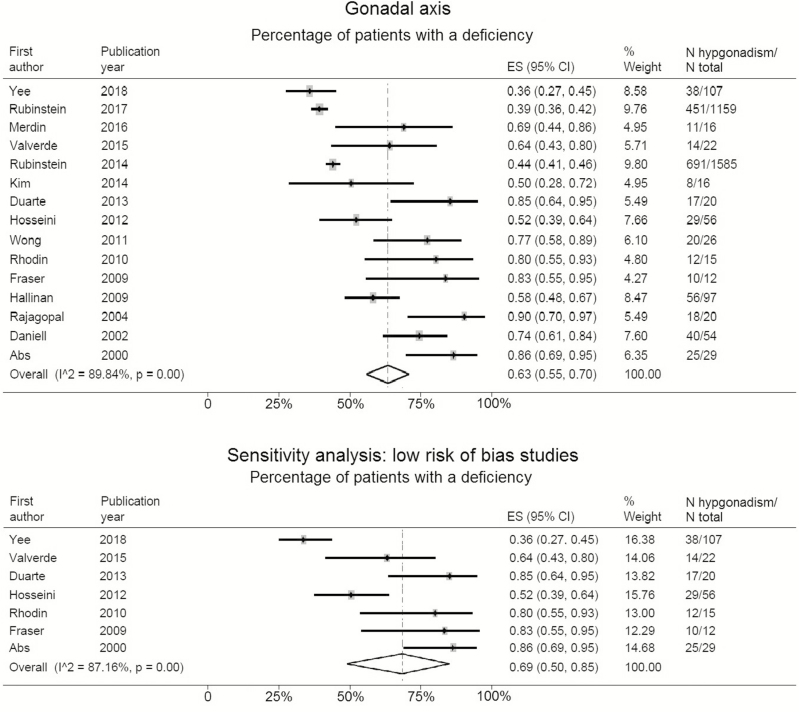
Pooled percentage and sensitivity analysis, only including low risk of bias studies, of opioid exposure-related hypogonadism. It is important to note that 99.5% of the analyzed patients were males.

Fifteen studies that included a total of 3250 patients presented data on the percentage of patients with hypogonadism among chronic opioid users, based on a single (morning or random) testosterone measurement (Table 1). Of the analyzed patients, 99.5% were males (*n* = 3234). The percentage of patients with hypogonadism ranged between 36% and 100%, with a weighted mean percentage of 63% (95% confidence interval [CI]: 55%–70%). Sensitivity analysis, among 7 studies with a low risk of bias, showed hypogonadism among 69% of male patients (95% CI: 50%–85%) (Fig. 2).

**Table 1.** Characteristics of the studies included in the meta-analyses

	Gonadal axis		Cotricotropic axis	
Number of studies	Total (*n* = 15)	Missing	Total (*n* = 5)	Missing
Total number of participants	3250	0	207	0
Median number of participants per study (range)	38 (12–1585)	0	48 (20–81)	0
Median % male (range)	100 (36–100)	1	52 (40–65)	1
Median mean age (range), years	50 (38–60)	2	50 (43–54)	1
Median duration of opioid use (range), months	26.6 (6–100)	8	18 (0–27)	2
Median long-term follow-up, months	9 (0–27)	11	9 (0–27)	1

**Figure 3.**
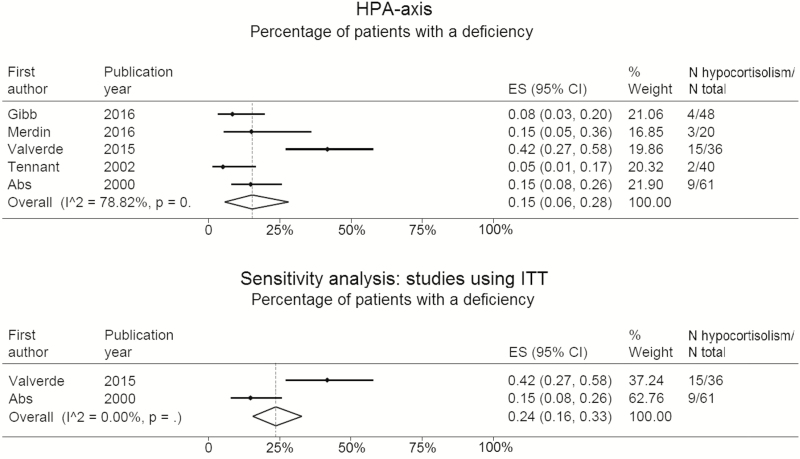
Pooled percentage and sensitivity analysis, only including studies using ITT as assessment of the HPA axis, of opioid exposure-related hypocortisolism. Abbreviations: ITT, insulin tolerance test. HPA, hypothalamus–pituitary–adrenal.

Five studies presented data of 205 patients (58% male) with hypocortisolism (Table 1). The percentage of patients with hypocortisolism ranged from 5% to 42%, with a weighted mean percentage of 15% (95% CI: 6%–28%). Sensitivity analysis, including 2 studies performing an ITT, showed that 24% of patients (95% CI: 16%–33%) were classified as adrenal insufficient (Fig. 3).

### Systematic review of all hypothalamus–pituitary–end organ axes

#### Hypothalamo–pituitary–gonadal axis

All 27 studies (*n* = 16 256 patients) reported an inhibitory effect of opioids on the hypothalamic–pituitary–gonadal axis (20). The effects on the gonadal axis of each gender are reported separately.

##### Male hypothalamo–pituitary–gonadal axis.

 Most studies specifically evaluated gonadal function only in male patients on opioids (*n* = 15). Male patients on fentanyl, methadone, or oxycodone had increased odds for testosterone deficiency compared to those using hydrocodone (odds ratios [OR] 25.73, 7.33, and 3.15] (10). When comparing long-acting with short-acting opioids, patients on long-acting ones had higher odds (OR 3.39, 95% CI: 2.39–4.77) for testosterone deficiency (10). Another study showed hypogonadism among 57% (*n* = 351) of patients on long-acting opioids and among 35% (*n* = 340) of patients on short-acting opioids (22). Two studies reported on a dose-related pattern (22, 23). The odds for androgen deficiency were higher on high dose methadone (OR 1.16) than low dose (OR 1.01) as compared to controls (22). Moreover, total testosterone levels were lower in high-dose (172.1 ng/dL), intermediate-dose (188.5 ng/dL), and low-dose users (265.8 ng/dL) as compared to controls (449.1 ng/dL) (23). Regarding symptomatology, the results were limited. One study found no correlation between sexual dysfunction and opioid use (24), while others reported increased rates of male patients with reduced potency (25), lower sexual desire (26), erectile dysfunction (27) or with general sexual dysfunction (16) while on opioids. Specifically, impotence and erectile dysfunction were reported in 89% of opioid-using male patients (*n* = 48) (23). Importantly, 23 of 24 males (95.8%) recorded a rather sudden decrease and even disappearance of libido and potency shortly after initiating opioid administration in 1 study (7). Lastly, 50% of patients on morphine had osteopenia (T-score between –1.0 and –2.5 standard deviations [SD]), and 21.4% had osteoporosis (T-score at or below –2.5 SD) (28).

##### Female hypothalamo–pituitary–gonadal axis.

 Studies reporting specifically on the female hypothalamo–pituitary–gonadal axis are very limited (*n* = 2). Two studies found an inhibitory effect on serum total and free testosterone concentrations in women, while serum estradiol was not affected (24, 29). In 1 study, decreased libido and hot flashes were more frequent in women receiving morphine (25), while libido decreased or disappeared shortly after initiating opioid therapy in 67% (*n* = 22) in another (7). Amenorrhea was reported in 19% (*n* = 3) (25) and 67% (*n* = 14) (7) and irregular menses in 50% (*n* = 8) (25) and 33% (*n* = 7) (7) of opioid using premenopausal women in 2 studies, while menstrual cycle disorders were present in 87% of premenopausal women on long-term opioids (16). Another study reported that lower androgen levels were correlated to depressive symptoms in women on intrathecal opioids (27).

#### Hypothalamic–pituitary–adrenal axis

In total, 21 studies with 1095 patients described the effects of opioid use on the activity of the HPA axis (20). Of these, 9 reported an inhibitory effect, 4 a stimulatory effect, and 8 studies reported no effect (12–14, 30–33). Evaluation of the HPA axis was performed in most studies by measurement of nonstimulated salivary, or serum cortisol (*n* = 12). A stimulation test was used in 8 of the studies (*n* = 208) of which 2 used the ITT; 2 the adrenocorticotropic hormone (ACTH) stimulation test; 1 the corticotropin-releasing hormone stimulation test; 1 the metyrapone test; and 1 yohimbine-stimulated cortisol. The 2 largest studies (*n* = 176 and *n* = 170) showed lower blood cortisol and ACTH levels in opioid users as compared to controls (12, 14). A dose-response relationship between opioid use and cortisol levels was reported by 2 studies, with a lower fasting cortisol (decrease of 8.6 nmol/L for every 10 mg morphine equivalent) (12) and a higher incidence of inadequate cortisol response to ACTH stimulation with higher dosages of opioids (0% on low-dose vs. 9% [*n* = 3] on high-dose) (13). The 2 studies using the ITT reported inadequate cortisol peaks 22% (*n* = 4) of the control subjects compared to 33% (*n* = 6) of the patients on intrathecal opioids and in 50% (*n* = 9) of those on oral opioids (25), and 15% of the opioid users had an insufficient cortisol response (7).

Clinical data on HPA axis morbidity in opioid users is very limited to absent. Abs et al. (7) did report a patient with HPA axis insufficiency that developed symptomatology of an Addisonian crisis during an episode of fever due to pneumonia. The patient recovered with supplementation of corticosteroids.

One study using a health status questionnaire showed that chronic pain patients with opioid-induced hypocortisolism offered low-dose hydrocortisone replacement reported better scores on vitality and pain compared with the placebo group (34).

#### Hypothalamic–pituitary–thyroid axis

Seven studies, including 274 patients, described the results of the effects of opioids on the HPT axis (20). One study showed that higher TSH levels were seen after acute administration of morphine compared to those prior to administration (15). Additionally, higher TSH levels following thyrotropin-releasing hormone (TRH) stimulation were found among patients on long-term opioids compared to healthy controls (16). One study found lower serum fT4 values among high-dose kratom (opioid tea; >3 glasses daily) users compared to low-dose users (≤3 glasses daily; 14.3 vs. 16.2mU/L) (35). Another study found lower serum fT4 in 6 out of 19 (32%) patients on intrathecal opioids and in 6 out of 18 (33%) patients on oral opioids, compared to 0 (0%) healthy controls (25).

#### Prolactin secretion

Seven studies (*n* = 354 patients) reported results on the effects of opioids on prolactin secretion (20). Four studies showed an increase in serum prolactin levels among patients on opioid analgesics (7, 16, 24, 30), whereas another study reported that 40% of patients (*n* = 8) had hyperprolactinemia, and all other patients had normal prolactin levels (8).

#### Somatotropic axis

Five studies described the effect of opioids on the somatotropic axis among 234 patients (20). One study showed lower serum insulin-like growth factor 1 (IGF-1) among 71 intrathecal opioid users compared to 20 controls (138.5 vs. 162.0 µg/L) and a lower GH peak during an ITT (14.5 vs. 20.9 µg/L) (7). In another study, a low ITT GH peak (<3.2 ng/mL) was seen in 2 subjects on intrathecal opioids (12%) versus none on oral opioids and 1 control subject (6%) (25).

#### Testosterone replacement

Six studies described the effect of testosterone replacement among 280 patients with opioid-related hypogonadism (20). Of these, 5 studies demonstrated an increase in serum testosterone following testosterone administration (36–40). Patients on testosterone replacement reported improved sexual function (36, 40, 41), sexual desire (37), and mental quality of life (36) as compared to placebo. One study showed that patients with opioid exposure-related hypogonadism who received testosterone replacement had a lower reduction of bone mineral density (T-scores) compared to patients receiving placebo (–0.73 [SD 0.13] vs. –1.61 [SD 0.23]) (38).

## Discussion

The results of this meta-analysis indicate that hypogonadism is present among approximately 63% of male patients on chronic opioids, while hypocortisolism is present in 15% to 24% of patients of both genders. In addition, hyperprolactinemia was a common feature in chronic opioid users. No definite conclusions can be drawn on the effects on the somatotropic and HPT axes.

Our results are in line with other smaller reviews, reporting on the effects of opioids on the endocrine system (42–47). As nearly all studies on opioid exposure-related hypogonadism included male patients, a definitive conclusion on the prevalence of opioid exposure-related hypogonadism among female patients cannot be drawn. It is plausible to assume that opioid exposure-related hypogonadism may also be present in women, as opioids suppress gonadal hormone secretion in both animals and humans via a central mechanism (11). Besides the inhibitory effect of opioids on the gonadal axis, our study showed that the likelihood of gonadotroph deficiency differed between the various opioids, and this was highest after fentanyl exposure. The likelihood of developing hypogonadism also appeared to increase when long-acting opioids or higher dosages were used.

In addition to the effects on the gonadal axis, we also found an inhibitory effect of opioids on cortisol levels. This is of particular interest, as untreated adrenal insufficiency can result in severe morbidity and, in case of an untreated Addisonian crisis, even death. Patients with diagnosed adrenal insufficiency receive higher stress doses of glucocorticoid replacement in stressful circumstances. Some of these circumstances are illness and severe pain. As opioids are mainly subscribed in cancer pain and noncancer pain patients, missing the diagnosis of adrenal insufficiency can be particularly harmful. In contrast to our findings, two studies described higher ACTH concentrations, and (hair) cortisol in opioid-treated patients compared to controls (16, 17). The higher levels of ACTH might be caused by increased chronic stress, anxiety, and depression among patients who were on methadone maintenance treatment since it has been shown that patients on methadone maintenance treatment had higher levels of ACTH compared to controls (17). In accord with our results, a study comparing long-term opioid users to age- and sex-matched controls found that 22.5% of the opioid users failed on an ACTH or metyrapone stimulation test, with a higher incidence of insufficiency with higher dosages of opioids, whereas test results were normal in all controls (48). Regarding quality of life, opioid users reported a worse quality of life compared to controls on the physical, social, and emotional role functioning; bodily pain; vitality; and mental health domains of the Short Form 36 (SF-36). The inhibitory effect of opioids on the gonadal and HPA axes is reported to be reversible when the opioid dose is tapered or when opioid therapy is abrogated (2).

There is insufficient evidence for a clear effect of opioids on the activity of the HPT and somatotropic axes. Animal studies show that acute administration of morphine increases TSH levels after stimulation with TRH (2). Devilla et al. (15) reported a prompt increase in TSH after morphine administration in humans as well. However, amongst chronic opioid users no difference has been found in TSH or fT4 levels compared to controls (2). Regarding the somatotropic axis, animal studies showed stimulated GH secretion after short-term administration of opioids (49). However, after long-term administration of opioids, there was no difference in IGF-1 levels or in GH secretion during dynamic testing (50).

Data on the interaction between the different axes are lacking. This may be of interest as the gonadal axis is impaired and prolactin levels are raised in opioid users. As prolactin inhibits GnRH release from the hypothalamus, this is an additional mechanism of the hypogonadism observed in patients on opioids (9).

### Strengths and limitations

A major strength of this study is that this is the first study performing a meta-analysis on this topic. This provides a more accurate estimation of the percentage of opioid exposure-related hypogonadism and cortisol deficiency, compared to narrative reviews. Our results were supported by the sensitivity analysis, which yielded similar results as those of the main analysis. The studies in this sensitivity analyses have been mainly selected on the most reliable endocrine testing being used. These analyses resulted in prevalence in the upper range of what has been previously reported in reviews (2, 9, 51) and stress the importance of thorough endocrinological examination of opioid users. A further strength is our comprehensive approach, as this is the first systematic review that assesses all relevant studies on the activity of the gonadal, HPA, HPT, and somatotropic axes and on prolactin secretion. Therefore, we were able to provide a better understanding of what is known and unknown on this topic. Moreover, we have included studies looking at treatment with hormone substitution therapy and its effects on the relevant symptomatology. This study has demonstrated absence of data on the female gonadal, the somatotroph and thyrotroph axes. Moreover, we report significant lack of clinical data on this field.

Our study does have some limitations. While we included studies on the effects of opioid use on all pituitary axes, we were able to analyze only the results on the effects on testosterone and cortisol secretion through meta-analysis, due to the limited number of studies reporting on the percentage of patients with endocrine effects for the other hypothalamo–pituitary–end hormone axes. Furthermore, the heterogeneity between studies may have affected the robustness of our results. This heterogeneity is most prominent in the type of opioid used and the included study population between studies. For example, the effect of methadone on the gonadotropic axis seems to be larger than the effect of buprenorphine in several studies comparing the two (26, 52, 53). Although most reports were in pain patients (both cancer and noncancer), there were also studies on patients on maintenance treatment for addiction, patients addicted to recreational opioids (heroin, kratom), and healthy volunteers. However, the results of our systematic review showed no difference in the effects of opioids on the endocrine system between different populations. Additionally, the duration of opioid exposure, the method of endocrine assessments, and definitions of endocrine deficiency differed between studies, which may affect the reported outcomes. Although not possible for this meta-analysis due to the heterogeneity of endocrine assessments, studies with the same endocrine assessments and same cut-off values should preferably be analyzed together to obtain more homogenous results. Also, because of the limited number of longitudinal studies, an interaction of the different endocrine axes could not be assessed. Finally, publication bias cannot be excluded, especially in intervention studies.

### Clinical implications and future research

Based on the results of this meta-analysis, periodic evaluation of the gonadal axis in males and adrenal axis is advisable when patients are exposed to long-term exogenous opioids, and this should be included in international guidelines on opioid use. Future studies should focus on the relationship between biochemical alterations indicating possible hormone deficiencies and experienced symptoms, using, among others, patient reported outcomes which measure the actual impact on patients’ lives in parallel to the time course of this symptomatology. Additionally, the added value of screening for these deficiencies and their possible treatment should be assessed, as with the opioid epidemic we currently face, this might pose a tremendous (economic) burden on health-care systems worldwide. Attention should also be given to hypogonadism in female patients, the HPT and somatotropic axes, and prolactin secretion in both sexes, as we currently lack data on these areas.

## Additional Information

***Disclosure Summary:*** The authors report no conflict of interest.

***Data Availability:*** Search strategy, MOOSE checklist, a list of all included articles, risk of bias assessment guide, results of the risk of bias assessment, and data supplemental tables are made available in a data repository (20).

## References

[1] Dart RC, Surratt HL, Cicero TJ, Parrino MW, Severtson SG, Bucher-Bartelson B, Green JL Trends in opioid analgesic abuse and mortality in the United States. N Engl J Med. 2015;372(3):241–248. 25587948 10.1056/NEJMsa1406143

[2] Fountas A, Chai ST, Kourkouti C, Karavitaki N Mechanisms in endocrinology: endocrinology of opioids. Eur J Endocrinol. 2018. [Epub ahead of print] doi:10.1530/EJE-18-0270 10.1530/EJE-18-0270 30299887

[3] Blendon RJ, Benson JM The public and the opioid-abuse epidemic. N Engl J Med. 2018;378(5):407–411. 29298128 10.1056/NEJMp1714529

[4] Volkow ND, McLellan AT Opioid abuse in chronic pain: misconceptions and mitigation strategies. N Engl J Med. 2016;374(13):1253–1263. 27028915 10.1056/NEJMra1507771

[5] Edgerton L, Loven B What are the adverse effects of prolonged opioid use in patients with chronic pain? J Fam Pract. 2011;60(5):288–289. 21544277

[6] Noble M, Treadwell JR, Tregear SJ, Coates VH, Wiffen PJ, Akafomo C, Schoelles KM Long-term opioid management for chronic noncancer pain. Cochrane Database Syst Rev. 2010(1):Cd006605. 20091598 10.1002/14651858.CD006605.pub2 PMC6494200

[7] Abs R, Verhelst J, Maeyaert J, Van Buyten JP, Opsomer F, Adriaensen H, Verlooy J, Van HT, Smet M, Van AK Endocrine consequences of long-term intrathecal administration of opioids. J Clin Endocrinol Metab. 2000;85(6):2215–2222. 10852454 10.1210/jcem.85.6.6615

[8] Merdin A, Merdin FA, Gunduz S, Bozcuk H, Coskun HS Opioid endocrinopathy: a clinical problem in patients with cancer pain. Exp Ther Med. 2016;11(5):1819–1822. 27168810 10.3892/etm.2016.3156 PMC4840782

[9] Coluzzi F, Billeci D, Maggi M, Corona G Testosterone deficiency in non-cancer opioid-treated patients. J Endocrinol Invest. 2018;41(12):1377–1388. 30343356 10.1007/s40618-018-0964-3 PMC6244554

[10] Rubinstein AL, Carpenter DM Association between commonly prescribed opioids and androgen deficiency in men: a retrospective Cohort analysis. Pain Med. 2017;18(4):637–644. 27516365 10.1093/pm/pnw182 PMC5410969

[11] Katz N, Mazer NA The impact of opioids on the endocrine system. Clin J Pain. 2009;25(2):170–175. 19333165 10.1097/AJP.0b013e3181850df6

[12] Peeters B, Guiza F, Boonen E, Meersseman P, Langouche L, Van den Berghe G Drug-induced HPA axis alterations during acute critical illness: a multivariable association study. Clin Endocrinol. 2017;86(1):26–36. 10.1111/cen.13155 27422812

[13] Gibb FW, Stewart A, Walker BR, Strachan MW Adrenal insufficiency in patients on long-term opioid analgesia. Clin Endocrinol (Oxf). 2016;85(6):831–835. 27260138 10.1111/cen.13125

[14] Aloisi AM, Buonocore M, Merlo L, Galandra C, Sotgiu A, Bacchella L, Ungaretti M, Demartini L, Bonezzi C Chronic pain therapy and hypothalamic–pituitary–adrenal axis impairment. Psychoneuroendocrinology. 2011;36(7):1032–1039. 21256679 10.1016/j.psyneuen.2010.12.017

[15] Devilla L, Pende A, Morgano A, Giusti M, Musso NR, Lotti G Morphine-induced TSH release in normal and hypothyroid subjects. Neuroendocrinology. 1985;40(4):303–308. 3921862 10.1159/000124091

[16] Rhodin A, Stridsberg M, Gordh T Opioid endocrinopathy: a clinical problem in patients with chronic pain and long-term oral opioid treatment. Clin J Pain. 2010;26(5):374–380. 20473043 10.1097/AJP.0b013e3181d1059d

[17] Yang J, Li J, Xu G, Zhang J, Chen Z, Lu Z, Deng H Elevated hair cortisol levels among heroin addicts on current methadone maintenance compared to controls. PLoS One. 2016;11(3):e0150729. 27010803 10.1371/journal.pone.0150729 PMC4806835

[18] Moher D, Liberati A, Tetzlaff J, Altman DG, Group P Preferred reporting items for systematic reviews and meta-analyses: the PRISMA statement. PLoS Med. 2009;6(7):e1000097. 19621072 10.1371/journal.pmed.1000097 PMC2707599

[19] Stroup DF, Berlin JA, Morton SC, Olkin I, Williamson GD, Rennie D, Moher D, Becker BJ, Sipe TA, Thacker SB Meta-analysis of observational studies in epidemiology: a proposal for reporting. Meta-analysis of observational studies in epidemiology (MOOSE) group. JAMA. 2000;283(15):2008–2012. 10789670 10.1001/jama.283.15.2008

[20] de Vries F, Bruin M, Lobatto DJ, et al Supplementary data to the paper: Opioids and their endocrine effects. 4TU.Centre for Research Data. Deposited 29 July 2019. 10.4121/uuid:983ea5a3-3914-4fad-814a-f7af41ddbf43.

[21] Nyaga VN, Arbyn M, Aerts M Metaprop: a Stata command to perform meta-analysis of binomial data. Arch Public Health. 2014;72(1):39. 25810908 10.1186/2049-3258-72-39 PMC4373114

[22] Rubinstein A, Carpenter DM Elucidating risk factors for androgen deficiency associated with daily opioid use. Am J Med. 2014;127(12):1195–1201. 25063648 10.1016/j.amjmed.2014.07.015

[23] Daniell HW Hypogonadism in men consuming sustained-action oral opioids. J Pain. 2002;3(5):377–384. 14622741 10.1054/jpai.2002.126790

[24] Wong D, Gray DP, Simmonds M, Rashiq S, Sobolev I, Morrish DW Opioid analgesics suppress male gonadal function but opioid use in males and females does not correlate with symptoms of sexual dysfunction. Pain Res Manag. 2011;16(5):311–316. 22059201 10.1155/2011/807123 PMC3206779

[25] Valverde-Filho J, da Cunha Neto MB, Fonoff ET, Meirelles ES, Teixeira MJ Chronic spinal and oral morphine-induced neuroendocrine and metabolic changes in noncancer pain patients. Pain Med. 2015;16(4):715–725. 25521923 10.1111/pme.12661

[26] Yee A, Loh HS, Danaee M, Riahi S, Ng CG, Sulaiman AH Plasma testosterone and sexual function in southeast asian men receiving methadone and buprenorphine maintenance treatment. J Sex Med. 2018;15(2):159–166. 29275046 10.1016/j.jsxm.2017.12.004

[27] Kim CH, Garcia R, Stover J, Ritchie K, Whealton T, Ata MA Androgen deficiency in long-term intrathecal opioid administration. Pain Physician. 2014;17(4):E543–E548. 25054405

[28] Duarte RV, Raphael JH, Southall JL, Labib MH, Whallett AJ, Ashford RL Hypogonadism and low bone mineral density in patients on long-term intrathecal opioid delivery therapy. BMJ Open. 2013;3(6):e002856. 10.1136/bmjopen-2013-002856 PMC3669726 23794541

[29] Daniell HW Opioid endocrinopathy in women consuming prescribed sustained-action opioids for control of nonmalignant pain. J Pain. 2008;9(1):28–36. 17936076 10.1016/j.jpain.2007.08.005

[30] Ambrosi B, Bochicchio D, Faglia G Loperamide, an opiate analogue, inhibits plasma ACTH levels in patients with Addison’s disease. Clin Endocrinol. 1986;24(5):483–489. 10.1111/j.1365-2265.1986.tb03276.x 3024866

[31] Auernhammer CJ, Riepl RL, Schopohl J, Lehnert P, Muller OA, Stalla GK In man the mu-opiate agonist loperamide specifically inhibits ACTH secretion induced by the cholecystokinin-like peptide ceruletide. Neuroendocrinology. 1994;60(1):16–22. 8090278 10.1159/000126715

[32] Zhang GF, Ren YP, Sheng LX, Chi Y, Du WJ, Guo S, Jiang ZN, Xiao L, Luo XN, Tang YL, Smith AK, Liu ZQ, Zhang HX Dysfunction of the hypothalamic-pituitary-adrenal axis in opioid dependent subjects: effects of acute and protracted abstinence. Am J Drug Alcohol Abuse. 2008;34(6):760–768. 19016181 10.1080/00952990802385781

[33] Gerber H, Borgwardt SJ, Schmid O, Gerhard U, Joechle W, Riecher-Rossler A, Wiesbeck GA, Walter M The impact of diacetylmorphine on hypothalamic–pituitary–adrenal axis activity and heroin craving in heroin dependence. Eur Addict Res. 2012;18(3):116–123. 22286020 10.1159/000334411

[34] Nenke MA, Haylock CL, Rankin W, Inder WJ, Gagliardi L, Eldridge C, Rolan P, Torpy DJ Low-dose hydrocortisone replacement improves wellbeing and pain tolerance in chronic pain patients with opioid-induced hypocortisolemic responses: a pilot randomized, placebo-controlled trial. Psychoneuroendocrinology. 2015;56:157–167. 25827960 10.1016/j.psyneuen.2015.03.015

[35] Singh D, Murugaiyah V, Hamid SBS, Kasinather V, Chan MSA, Ho ETW, Grundmann O, Chear NJY, Mansor SM Assessment of gonadotropins and testosterone hormone levels in regular Mitragyna speciosa (Korth.) users. J Ethnopharmacol. 2018;221:30–36. 29626673 10.1016/j.jep.2018.04.005

[36] Aloisi AM, Ceccarelli I, Carlucci M, Suman A, Sindaco G, Mameli S, Paci V, Ravaioli L, Passavanti G, Bachiocco V, Pari G Hormone replacement therapy in morphine-induced hypogonadic male chronic pain patients. Reprod Biol Endocrinol. 2011;9:26. 21332999 10.1186/1477-7827-9-26 PMC3049183

[37] Basaria S, Travison TG, Alford D, Knapp PE, Teeter K, Cahalan C, Eder R, Lakshman K, Bachman E, Mensing G, Martel MO, Le D, Stroh H, Bhasin S, Wasan AD, Edwards RR Effects of testosterone replacement in men with opioid-induced androgen deficiency: a randomized controlled trial. Pain. 2015;156(2):280–288. 25599449 10.1097/01.j.pain.0000460308.86819.aa PMC6036339

[38] Finch PM, Price LM, Pullan PT, Drummond PD Effects of testosterone treatment on bone mineral density in hypogonadal men receiving intrathecal opioids. Pain Pract. 2015;15(4):308–313. 24690205 10.1111/papr.12190

[39] Huang G, Travison TG, Edwards RR, Basaria S Effects of testosterone replacement on pain catastrophizing and sleep quality in men with opioid-induced androgen deficiency. Pain Medicine. 2017;18(6):1070–1076. 27550959 10.1093/pm/pnw159

[40] Raheem OA, Patel SH, Sisul D, Furnish TJ, Hsieh TC The role of testosterone supplemental therapy in opioid-induced hypogonadism: a retrospective pilot analysis. Am J Mens Health. 2017; 11(4):1208–1213. 28625114 10.1177/1557988316672396 PMC5675327

[41] Blick G, Khera M, Bhattacharya RK, Nguyen D, Kushner H, Miner MM Testosterone replacement therapy outcomes among opioid users: the Testim Registry in the United States (TRiUS). Pain Med. 2012;13(5):688–698. 22536837 10.1111/j.1526-4637.2012.01368.x

[42] Gudin JA, Laitman A, Nalamachu S Opioid related endocrinopathy. Pain Med. 2015;16(Suppl 1):S9–S15. 26461076 10.1111/pme.12926

[43] McWilliams K, Simmons C, Laird BJ, Fallon MT A systematic review of opioid effects on the hypogonadal axis of cancer patients. Support Care Cancer. 2014;22(6):1699–1704. 24633592 10.1007/s00520-014-2195-2

[44] Brennan MJ The effect of opioid therapy on endocrine function. Am J Med. 2013;126(3 Suppl 1):S12–S18. 23414717 10.1016/j.amjmed.2012.12.001

[45] Vigano A, Piccioni M, Trutschnigg B, Hornby L, Chaudhury P, Kilgour R Male hypogonadism associated with advanced cancer: a systematic review. Lancet Oncol. 2010;11(7):679–684. 20541464 10.1016/S1470-2045(10)70021-8

[46] Merza Z Chronic use of opioids and the endocrine system. Horm Metab Res. 2010;42(9):621–626. 20486065 10.1055/s-0030-1254099

[47] Bawor M, Bami H, Dennis BB, Plater C, Worster A, Varenbut M, Daiter J, Marsh DC, Steiner M, Anglin R, Coote M, Pare G, Thabane L, Samaan Z Testosterone suppression in opioid users: a systematic review and meta-analysis. Drug and Alcohol Depend. 2015;149:1–9. 10.1016/j.drugalcdep.2015.01.038 25702934

[48] Lamprecht A, Sorbello J, Jang C, Torpy DJ, Inder WJ Secondary adrenal insufficiency and pituitary dysfunction in oral/transdermal opioid users with non-cancer pain. Eur J Endocrinol. 2018;179(6):353–362. 30324794 10.1530/EJE-18-0530

[49] Bartolome MB, Kuhn CM Endocrine effects of methadone in rats; acute effects in adults. Eur J Pharmacol. 1983;95(3–4):231–238. 6653672 10.1016/0014-2999(83)90639-8

[50] Armstrong JD, Spears JW Changes in growth hormone and luteinizing hormone following acute or chronic administration of an opioid agonist, FK33-824, in wethers. J Anim Sci. 1991;69(2): 774–781. 2016203 10.2527/1991.692774x

[51] Donegan D, Bancos I Opioid-induced adrenal insufficiency. Mayo Clin Proc. 2018;93(7):937–944. 29976376 10.1016/j.mayocp.2018.04.010

[52] Hallinan R, Byrne A, Agho K, McMahon CG, Tynan P, Attia J Hypogonadism in men receiving methadone and buprenorphine maintenance treatment. Int J Androl. 2009;32(2):131–139. 17971165 10.1111/j.1365-2605.2007.00824.x

[53] Bliesener N, Albrecht S, Schwager A, Weckbecker K, Lichtermann D, Klingmuller D Plasma testosterone and sexual function in men receiving buprenorphine maintenance for opioid dependence. J Clin Endocrinol Metab. 2005;90(1):203–206. 15483091 10.1210/jc.2004-0929

